# Jean Aicardi (1926–2015)

**DOI:** 10.1007/s00415-024-12684-8

**Published:** 2024-09-10

**Authors:** Alisha Huang, Saba Jafarpour, Nicole A. Nishimori, Lilia Kazerooni, Jonathan D. Santoro

**Affiliations:** 1 University of Southern California, Los Angeles, CA France; 2 Division of Neurology, Department of Pediatrics, Children’s Hospital Los Angeles, 4650 Sunset Blvd, Mailstop 82, Los Angeles, CA 90027 USA; 3 Department of Neurology, Keck School of Medicine of the University of Southern California, Los Angeles, CA France

**Fig. 1:**
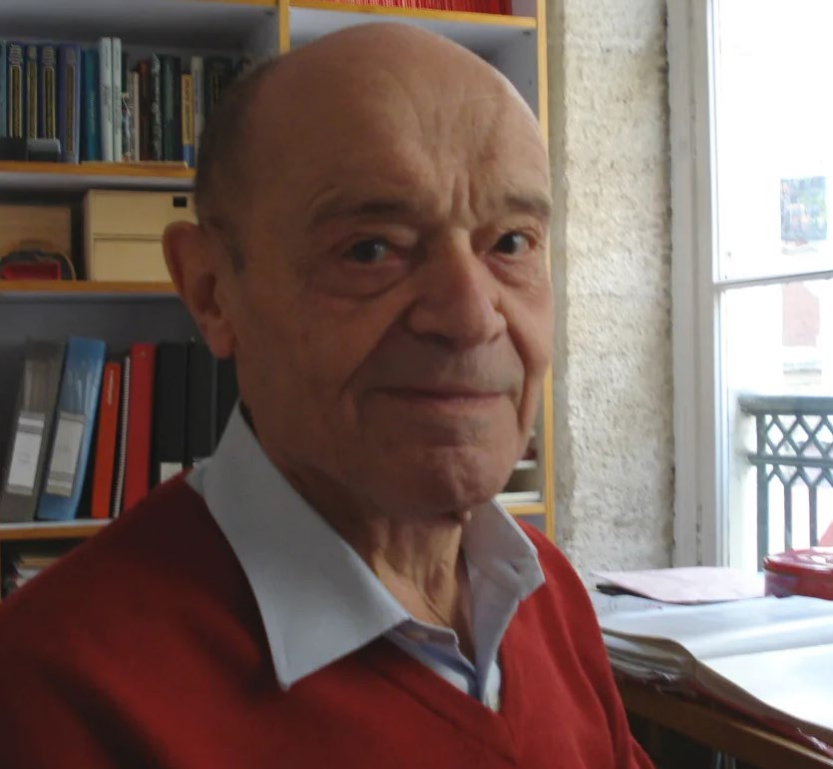
Jean François Marie Aicardi (1926–2015)

Jean François Marie Aicardi (Fig. 1) was born on November 8, 1926 as the seventh child in a tight-knit, hardworking family in Rambouillet, 50 km from Paris, France. He attended the Lycée Hoche in Versailles, where he felt indifferent toward most subjects—particularly mathematics—but found himself fascinated by biology. This interest gradually manifested into an earnest aspiration of pursuing a career in medicine, with his first preparatory year at the Faculté des Sciences de l’Université de Paris, solidifying Aicardi’s passion for the natural sciences—despite growing up with parents working in non-medical fields like radiotelegraphy [1].

While a medical student, he trained under neurology professor Raymond Garcin, who brought about Aicardi’s love for clinical neurology. In 1955, Aicardi obtained his medical degree from the Faculté de Médecine in Paris and successfully passed the Concours d’Internat examination needed to pursue his clinical academic career. His rewarding and exciting experience as an intern at Hôpital des Enfants Malades in professor Stéphane Thieffry’s Unit, the first French child neurology service, consisted of patient diagnostic work and treatment, which is what ultimately made Aicardi realize his keen enthusiasm for pediatric neurology. Aicardi completed a 2-year research fellowship in pediatric neurology at Boston Children’s Hospital in 1955–1956, working with Randolph Byers, Cesare Lombroso, and Giuseppe Erba. After completing his training and gaining an understanding on the differences between health systems in the United States and France, he repatriated in 1956; in addition to being a medical assistant at the Surgical Service at Hôpital des Enfants Malades and working in public outpatient pediatric dispensaries to provide for his wife and himself, Aicardi took on an unofficial position at the Thieffry Unit, where he engaged in teaching and tended to children with nervous conditions [1].

Aicardi served as Director of Research for the Thieffry Unit at the Hôpital des Enfants Malades, the Hôpital Saint-Vincent-de-Paul, and France’s National Institute of Health and Medical Research (INSERM), from 1969 to 1991. There, he was instrumental in the systematic reporting of multiple rare neurogenetic diseases. Aicardi went onto becoming an eponym of two rare conditions, Aicardi syndrome and Aicardi-Goutières syndrome, and discovered Rett syndrome, alternating hemiplegia of infancy, and delayed encephalitides following rubeola—among other diseases [1].

Owing to Henri Gastaut’s burgeoning interest in epilepsy, Aicardi frequently collaborated with epilepsy investigator Jean-Jacques Chevrie and published a series of articles on convulsive disorders, childhood epilepsy, and Aicardi syndrome (AS) [1]. While AS was first identified by Aicardi in 1965 as a disorder characterized by callosal agenesis, infantile spasm in flexion, and ophthalmic abnormalities, the attributes of this syndrome were further refined in an in-depth study by Aicardi and his colleague Chevrie by examining 184 clinical cases in 1986 [2]. Aicardi and Chevrie made notable discoveries regarding features of AS like exclusive prevalence in women, partial or incomplete agenesis of the corpus callosum, absence of familial recurrence, chorioretinal lacunae abnormalities, microphthalmia, coloboma of the optic disk, severe mental retardation, subependymal heterotopias, abnormal vertebrae and ribs, and ‘split-brain’ electroencephalography (EEG) showing a burst suppression pattern [2].

Together with Swedish researcher Bengt Hagberg at the Thieffry Neurological Unit, Aicardi also made important strides in the characterization of the newly discovered, yet overlooked Rett syndrome (RTT), a progressive encephalopathy which Aicardi and Hagberg correctly hypothesized to be an X-linked dominant disorder; in October 1983, when they reported findings on 35 women from France, Portugal, and Sweden who experienced developmental regression after the age of 7–18 months and subsequent rapid neurological deterioration [3]. By allowing their findings to be accessible in the English language, Hagberg and Aicardi drew attention to Andreas Rett’s primary identification of RTT and findings published in German in 1974 [3].

In 1984, Aicardi worked with Françoise Goutières to report the discovery of the novel Aicardi–Goutières syndrome (AGS), a genetically heterogeneous progressive encephalopathy inherited in an autosomal recessive manner [1]. AGS was observed by Goutières and Aicardi in 8 affected infants from 5 families with rare bilateral calcifications of the basal ganglia, atypical hypodensities of the white matter, and chronic CSF lymphocytosis [4]. Aicardi’s description of AGS was one of the original manuscripts on the interplay between neuroinflammatory disease and definitively neurogenetic syndromes. His initial reports formed the basis for multiple research studies on this condition with the recent advancement of the understanding that this condition, once thought to be untreatable, can be treated with immunotherapy [5].

In all, Aicardi was a pediatric neurologist and epileptologist of international renown, as evidenced by his numerous awards, including the Child Neurology Society John B. Hower Award (1986) as the first non-American recipient, the American Epilepsy Society Epilepsy Research Award (1995), and the Peter-Emil-Becker-Price Award (2002) [6, 7]. During his professional career, he published more than 400 articles, three books, including *Diseases of the Nervous System in Childhood*, a pediatric neurology classic, and founded the journal *Epileptic Disorders* (currently, *International League Against Epilepsy Educational Journal*) [8, 9]. Aicardi’s neurological endeavors extended beyond France, from being a visiting scientist at the Miami Children’s Hospital to conducting research and teaching at the Institutes of Child Health in London as Honorary Professor of Child Neurology after his retirement in the 1990s [8]. Throughout it all, Aicardi maintained an inspiring character of humility and never failed to express his gratitude to INSERM and fellow colleagues for motivating him to effect groundbreaking discoveries through the clinical lens [1]. In his final years, he grappled with tongue cancer. Aicardi passed away on the morning of August 3, 2015 [8, 10]. A trailblazer, a life-long learner, and an inspiration to all, Jean Aicardi left a permanent mark as a respected scientist in the fields of pediatric neurology and epilepsy.
