# Correction: Jean Aicardi (1926–2015)

**DOI:** 10.1007/s00415-024-12837-9

**Published:** 2025-01-09

**Authors:** Alisha Huang, Saba Jafarpour, Nicole A. Nishimori, Lilia Kazerooni, Jonathan D. Santoro

**Affiliations:** 1 University of Southern California, Los Angeles, CA USA; 2 Division of Neurology, Department of Pediatrics, Children’s Hospital Los Angeles, 4650 Sunset Blvd, Mailstop 82, Los Angeles, CA 90027 USA; 3 Department of Neurology, Keck School of Medicine of the University of Southern California, Los Angeles, CA USA

**Correction: Journal of Neurology (2024) 271:7363–7365** 10.1007/s00415-024-12684-8

In the original version of this article, affiliation 1 and 3 were incorrectly given as

^1^University of Southern California, Los Angeles, CA, France

^3^Department of Neurology, Keck School of Medicine of the University of Southern California, Los Angeles, CA, France

but should have been

^1^University of Southern California, Los Angeles, CA, USA

^3^Department of Neurology, Keck School of Medicine of the University of Southern California, Los Angeles, CA, USA

